# Probiotic Viability and Bioactive Properties of Buffalo Yoghurt Produced Using High Cholesterol‐Assimilating Probiotic Strains

**DOI:** 10.1002/vms3.70233

**Published:** 2025-02-06

**Authors:** Sinan Akbal, Esra Uğur Geçer, Pelin Ertürkmen

**Affiliations:** ^1^ Department of Food Processing Acıpayam Vocational School Pamukkale University Denizli Türkiye; ^2^ Faculty of Engineering Department of Food Engineering Süleyman Demirel University Isparta Türkiye; ^3^ Department of Food Processing Burdur Food, Agriculture and Livestock Vocational School Burdur Mehmet Akif Ersoy University Burdur Türkiye

**Keywords:** ACE‐inhibitory, antioxidant, buffalo yoghurt, cholesterol, probiotic culture

## Abstract

**Background:**

This study aimed to produce yoghurt with reduced cholesterol levels, enhanced antioxidant activity and angiotensin‐converting enzyme (ACE) inhibitory activity while maintaining acceptable health properties, using buffalo milk and probiotic microorganisms.

**Method:**

Buffalo yoghurts were produced using three different probiotic strains, including *Lactobacillus acidophilus*, *Lactiplantibacillus plantarum* and *Bifidobacterium lactis*. ACE‐inhibitor activities (%), antioxidant activities as DPPH (%), and cholesterol activities in HPLC of these yoghurts were determined during the 28‐day storage period. In addition, probiotic microorganisms, total aerobic mesophilic bacteria and yeast mould were counted during storage.

**Results:**

The viability of probiotic microorganisms in buffalo yoghurts remained above 5 log CFU/g at the end of the storage period. Antioxidant activity ranged from 9.30% to 27.20%. Buffalo yoghurt is produced with *Lpb. plantarum*, which exhibited the highest viability (9.12 log CFU/g) and antioxidant activity values of 61.48%. Gastrointestinal digestion affected the antioxidant and ACE‐inhibitor properties of the yoghurt samples. The highest ACE‐inhibitory effect after gastric digestion on the 28th day was observed in yoghurt‐produced *Lpb. plantarum* and *B. lactis*, with 24.30% and 25.14% values, respectively. Also, the ACE‐inhibitory activity of the outer (OUT) phase for all yoghurt samples was higher than that of undigested samples. According to cholesterol peaks obtained in HPLC, the highest cholesterol assimilation was detected in yoghurt produced using *Lpb. plantarum*.

**Conclusion:**

The data obtained from the study may contribute to research on the potential of probiotic microorganisms with cholesterol‐assimilation ability and probiotic food products produced using them to reduce cholesterol risk.

## Graphical abstract

Buffalo yoghurt samples containing *Lpb. plantarum* showed the highest probiotic activity.

*B. lactis* increased ACE‐inhibitory activity after gastrointestinal digestion.

Cholesterol levels are lower in buffalo yoghurts containing *Lpb. plantarum* and *B. lactis*. The data may contribute to research on probiotics and functional cultures and the development of new probiotic products.

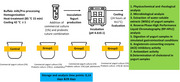

## 1 Introduction

Utilizing lactic acid bacteria (LAB) as probiotics presents an appealing method to reduce risk factors and provide multiple health benefits, particularly in dairy products. LAB, such as *Lactobacillus, Lactococcus, Pediococcus* and *Weissella* species, are commonly used as probiotic candidates (Derakhshan et al. 2023). They produce angiotensin‐converting enzyme (ACE) inhibitors and offer numerous health benefits for humans and animals in fermented foods, including dairy products, to down‐regulate hypertension (Jitpakdee et al. 2021; Song et al. 2022). Additionally, LAB provide antimicrobial, antioxidant, anticarcinogenic and anticholesterol effects (Asan‐Ozusaglam and Gunyakti 2019; Górska et al. 2019; Hao et al. 2021).

Safely isolated from fermented foods, LAB can enhance yoghurt's organoleptic and nutritional properties, making them suitable for commercial applications. Using *Lacticaseibacillus rhamnosus* GG in yoghurt production increases the yield of volatile organic acids and alcohols during fermentation. It enhances the formation of non‐volatile organic acids and free amino acids during cold storage (Settachaimongkon et al. 2015). Another study reported that *Lacticaseibacillus casei* ATCC 393 increases the total phenolic substance and flavonoid content of yoghurt (Shori et al. 2022). Furthermore, yoghurts produced using probiotic microorganisms exhibit increased antioxidant and ACE‐inhibitory effects (Taha et al. 2017; Kim et al. 2021). It was also found that yoghurts produced with *Lactobacillus gasseri* 4/13, which has cholesterol assimilation properties, did not exhibit changes in taste and smell during 20 days of storage (Baltova and Dimitrov 2014). Another study reported that nine LAB isolates obtained from yoghurt have cholesterol‐lowering effects (Nurcahyani et al. 2023).

Cholesterol is an organic substance with many functions, including synthesizing vitamin D, bile acids and steroid hormones (Palaniyandi et al. 2020). Numerous researchers have established a significant relationship between cardiovascular diseases and high dietary cholesterol levels. Consequently, the consumption of cholesterol‐containing foods has decreased in recent years, and there is a growing trend towards the production of low‐cholesterol foods (Wang et al. 2019). Although many methods can reduce cholesterol levels, an interesting approach is using probiotic cultures in dairy production (Asan‐Ozusaglam and Gunyakti 2019; Fırıncıoğulları and Öner 2022; Chailangka et al. 2023).

Using buffalo milk in yoghurt production offers significant nutritional and sensory advantages. Despite having a higher fat content than cow milk, buffalo milk has a lower cholesterol level. This is attributed to the smaller diameter of fat globules and their richness in polyunsaturated fatty acids (Zicarelli 2004; Basilicata et al. 2018; Vargas‐Ramella et al. 2021). In producing buffalo yoghurt, there is a need for new probiotic strains with high functional properties and cholesterol‐assimilation capacity. Accordingly, *Lactiplantibacillus plantarum* isolated from cheese reduced cholesterol and was used in yoghurt production alongside other probiotic cultures (*Bifidobacterium lactis* and *Lactobacillus acidophilus*). The bioactive effects of yoghurts, such as ACE‐inhibitory effects, antioxidant activities and cholesterol levels, were examined before and after in vitro digestion. Additionally, the physicochemical and microbiological properties of yoghurts were compared during storage.

## 2 Materials and Methods

### 2.1 Probiotics and Buffalo Yoghurt Production

**FIGURE 1:**
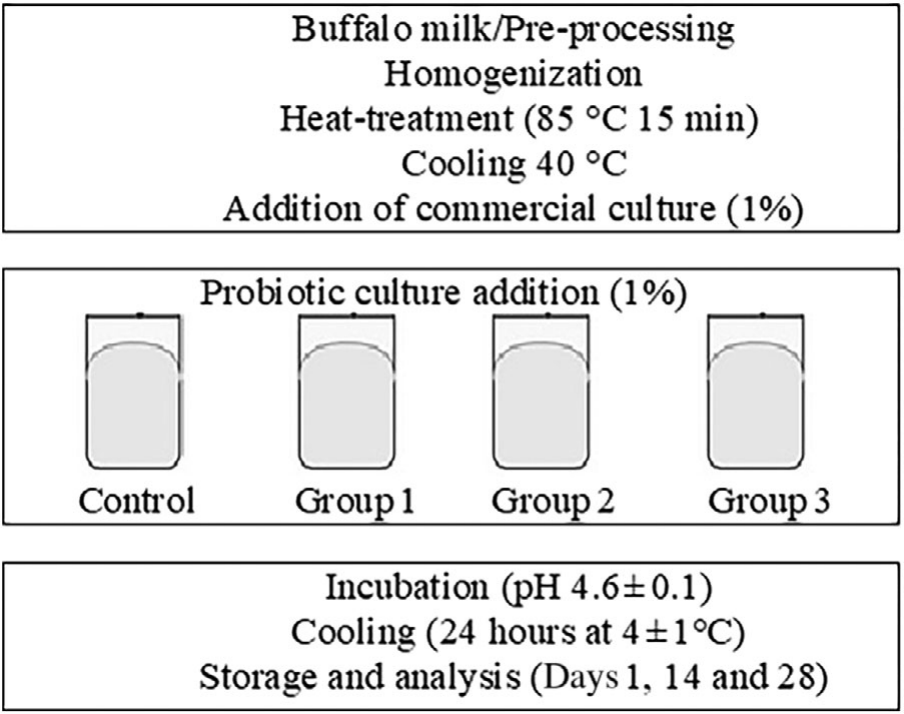
The pictorial representation of the probiotic buffalo yoghurt production. Control: commercial yoghurt culture (2%); Group 1: commercial yoghurt culture (1%) + probiotic culture (1%) (*L. acidophillus*); Group 2: commercial yoghurt culture (1%) + probiotic culture (1%) (*Lpb. plantarum*); Group 3: commercial yoghurt culture (1%) + probiotic culture (1%) (*B. lactis*).

The strain *Lpb. plantarum* Lb9 with high probiotic activity, from the culture collection of the Dairy Research Laboratory at Suleyman Demirel University's Food Engineering Department (Ertürkmen et al. 2023), along with two probiotic strains, *L. acidophilus* and *B. lactis*, from Burdur Mehmet Akif Ersoy University Food Technology Laboratory, were used as the materials. The buffalo milk used in buffalo yoghurt production and the commercial culture were sourced from the Milk Technologies Research and Development Center at Burdur Mehmet Akif Ersoy University. The probiotic strains, used in yoghurt production, were activated under anaerobic conditions using an anaerobic jar and Anaerocult A (Merck) kits for up to 24 h at 37°C in De Man, Rogosa, Sharpe (MRS) broth three times. They were then inoculated (2% inoculum, vol/vol) into 10% skimmed cow milk with 0.5% glucose and yeast extract and incubated at 37°C until curdled. Probiotic yoghurt production and names of the buffalo yoghurt groups are given in Figure 1.

### 2.2 Physicochemical and Rheological Analysis

During cold storage, pH, acidity percentage, dry matter, fat and protein analyses were performed on yoghurts. pH measurements were determined by immersing a pH electrode (Inolab WTW) in yoghurt at room temperature. The titratable acidity of the yoghurts was determined by titrating a 10 g sample with a 0.25 N NaOH (Merck) solution using phenolphthalein as an indicator (T.S.1330, Yoğurt Standardı. (Türk Standartları Enstitüsü) 1999). Protein analysis was performed using the macro Kjeldahl method (IDF 1993). The fat content of the samples was determined using the Gerber method (Öner and Aloğlu 2018). To determine the total dry matter content, the samples were dried in an oven at 105°C for 3–4 h until they reached a constant weight, and the results were calculated as a percentage (IDF 1987; AOAC 1990). For rheological analysis, the viscosity of buffalo milk yoghurt was measured following the method described by Şimşek and Gün (2021) using the No. 5 spindle of the Brookfield viscometer (Model DV‐II‐pro+; Brookfield Engineering Laboratories, Inc., MA, USA) at a speed of 10 rpm and a shear rate of 50 s^−1^ at 10°C for 60 s. Each result was recorded in centipoise (cP), and the average value of three measurements was taken (Al‐Shaikh et al. 2020).

### 2.3 Microbiological Analysis

Yoghurt samples were diluted using sterile peptone water (0.1%) to prepare appropriate dilutions up to 10^−8^. *Lactobacillus delbrueckii* subsp. bulgaricus enumeration was performed by inoculating these dilutions onto MRS Agar (Merck, Germany) and incubating them under anaerobic conditions at 43°C for 72 h. For *Streptococcus salivarius* subsp. *thermophilus*, the enumeration was conducted by plating on M‐17 Agar with an addition of 10% lactose (M17, Merck) under aerobic conditions at 37°C for 72 h (Ranasinghe and Perera 2016). *L. acidophilus* counts were determined by pour‐plating onto MRS Agar supplemented with 10% (w/v) d‐sorbitol under anaerobic conditions at 37°C for 72 h (Dave and Shah 1998). *Bifidobacterium* spp. counts were determined on MRS Agar (Merck, Germany) supplemented with 0.5 g/L cysteine (l‐Cysteine, Sigma‐Aldrich) under anaerobic conditions at 37°C for 48 h (Terzioğlu et al. 2023). Yeast and mould counts were conducted on Potato Dextrose Agar (PDA, Merck) acidified with 10% lactic acid and incubated at 25°C for 3–5 days. Total bacterial counts were enumerated using Plate Count Agar (PCA) (Biokar, France) and incubated at 30°C for 48 h. Enumeration results were expressed as log CFU/g (Karahan et al. 2002).

### 2.4 Extraction of Water‐Soluble Extracts of Yoghurt Samples

All yoghurt samples were diluted with pure water at a ratio of 1:2. Accordingly, 20 mL of the diluted samples were taken and kept in a water bath at 40°C for 30 min. Then, the pH of the samples was adjusted to 4.6 using 1 M HCl. Precipitated caseins were separated by centrifugation at 5000 rpm for 15 min at 4°C. The supernatant was filtered using Whatman No. 113 paper, yielding water‐soluble extracts (WSEs). For high‐performance liquid chromatography (HPLC) analysis, the WSE samples were mixed with pure water containing 0.2% TFA at a ratio of 1:1 and passed through a 0.45 µm cellulose acetate filter (Öner and Aloğlu 2018).

### 2.5 Reverse Phase‐HPLC Analysis of WSEs of Yoghurt Samples

WSEs were filtered through a 0.45‐µm filter and transferred to vials without lyophilization. Peptide analysis was conducted using RP‐HPLC with an Inertsil ODS‐4 (250 × 4.6 mm ID, 5 µm) C‐18 column. The analysis conditions were determined with some modifications based on the method Öner and Sarıdağ (2019) suggested. The mobile phase Solution A was 0.1% TFA in deionized water, and Solution B was 0.08% TFA in acetonitrile. The flow rate was 1.0 mL/min. Other analysis conditions included a 214 nm PDA detector, a column oven temperature of 20°C, and a running time of 100 min.

### 2.6 Digestion of Yoghurt Samples in Simulated Gastrointestinal Conditions

The method by McDougall et al. (2005) was employed in the gastrointestinal digestion of yoghurt samples. The AD phase samples were obtained to mimic gastric digestion during the digestion process. The part inside the dialysis tube (IN phase), which simulates digestion in the small intestine, represents the fraction absorbed from the small intestine into the bloodstream. The outer phase (OUT phase) represents the sample portion not absorbed in the small intestine (Uğur 2023). Five grams of yoghurt samples were weighed to simulate gastric digestion, and 7.5 mL of pepsin solution was added. Subsequently, 20 mL of 0.2% NaCl solution was added to each sample, and the pH was adjusted to 2 using 5 N HCl. The samples were then incubated in a shaking incubator at 37°C for 2 h at 110 rpm, resulting in the post‐digestion (AD) phase sample. After collecting the AD sample, intestinal digestion was simulated on the remaining sample. Accordingly, the remaining AD phase sample was added to 4.5 mL of intestinal fluid (18 mg pancreatin, 112.5 mg bile salt and 4.5 mL purified water). A dialysis tube (MWCO 12000 Da, SIGMA) containing sodium bicarbonate solution was placed in this mixture and incubated at 37°C at 110 rpm for 2 h. At the end of this period, the IN phase sample was collected from the dialysis bags, and the OUT phase sample was collected from the beaker. All digestion phase samples were centrifuged at 10,000 rpm for 15 min at 4°C, and the supernatant was stored at −18°C until analysis.

### 2.7 ACE‐Inhibitory Activity

To determine the ACE‐inhibitory activity, 20 µL of WSEs and digested yoghurt samples were transferred to an empty tube. Then, 100 µL of substrate solution (5 mM HHL in 0.1 M sodium borate buffer containing 0.3 M NaCl, pH 8.3) was added. Tube A, the control, was prepared by adding only 100 µL of substrate solution. Subsequently, 20 µL of ACE (0.1 U/mL) was added to the mixture. The mixture was incubated at 37°C for 30 min. After incubation, 1 mL of ethyl acetate was added to all tubes, and the top phase was transferred to another tube. The transferred liquid was then dried at 95°C for 20 min. Following this, 1 mL of distilled water was added to the dried tubes, and the absorbance was measured at 228 nm (Cushman and Cheung 1971; Meira et al. 2012). The ACE% inhibition was calculated using Equation (1), where A represents the control tube without the sample and B represents the tube containing the sample (Munir et al. 2020).

(1) $$\mathrm{ACE}-\mathrm{inhibitory}\mathrm{activity}\%=\left(A-B\right)/A\times 100$$

### 2.8 Antioxidant Activity

Antioxidant activity was determined for WSEs and digestion samples of yoghurt using the DPPH method (Aluko and Monu 2003; Farzamirad and Aluko 2008). To each tube, 1.5 mL of 0.1 M phosphate buffer solution (pH 7.0) containing 1% Triton X‐100 was added, followed by adding and mixing 200 µL of the sample. Subsequently, 1.5 mL of 100 µM DPPH radical solution prepared in methanol was added and stirred. The mixture was then kept in a dark environment at room temperature for 30 min. The absorbance was measured at a wavelength of 517 nm. The phosphate buffer solution was used as a blank. The percentage of antioxidant activity was determined using Equation (2).

(2) $$\begin{array}{ccc} \mathrm{Antioxidant}\;\mathrm{activity} & = & \left(\mathrm{Control}\;\mathrm{abs}-\mathrm{Sample}\;\mathrm{abs}\right)/\\ & & \left(\mathrm{Control}\;\mathrm{abs}\;\mathrm{value}\right)\times 100 \end{array}$$

### 2.9 Determination of Cholesterol in Yoghurt Samples

To determine the cholesterol values in the yoghurt samples, 2 g samples were weighed. Subsequently, 5 mL of 0.4 M KOH was added, and the mixture was vortexed for 1 min. The mixture was then incubated in a 50°C water bath for 30 min. After cooling to room temperature, 5 mL of ultrapure water was added, followed by vortexing for 1 min. Then, 10 mL of hexane was added, and after vortexing for 1 min, the mixture was allowed to undergo phase separation. The upper phase was collected into a flask. Another 10 mL of hexane was added, vortexed for 1 min and the separated upper phase was again collected. The phases were combined and evaporated in a rotary evaporator at 40°C. The residue was dissolved in 1 mL of the mobile phase.

The samples were analysed using Agilent liquid chromatography/mass spectrometry (LC/MS) with a Shimadzu LC‐20 AT HPLC system equipped with an SPD‐10Avp UV–vis detector (210 nm), consisting of an LC‐20 AD pump, a SIL‐20 AC autosampler, a CTO‐10 AS VP column oven and an LC‐20AT controller (Shimadzu, Kyoto, Japan). Cholesterol separation was performed using a Phenomenex Luna 5u C18 100 Å column (250  ×  4.6 mm ID) (Phenomenex, Torrance, CA, USA). The mobile phase (ACN/IPA, 70:30 v/v) was filtered through a 0.45‐µm membrane (Millipore, Bedford, MA, USA) and degassed for 30 min using a DGU‐20A5. The column and autosampler temperatures were maintained at 20°C, with a flow rate of 1.2 mL/min and a total run time of 15 min. A 10‐µL sample was injected into the chromatographic system. Detection was performed at 210 nm, with peak areas quantified and processed using Empower software version 2.0 (Waters, Milford, MA, USA). Cholesterol identification was achieved based on cholesterol standards' retention time and UV spectrum (Albuquerque et al. 2016).

### 2.10 Statistical Analysis

Statistical analysis was conducted using one‐way ANOVA in Minitab 17 to assess the differences between groups in the analysed buffalo yoghurt samples. The differences between cheese samples during ripening were evaluated using Tukey's multiple comparison test, with a significance level set at *p* < 0.05.

## 3 Results

### 3.1 Physicochemical and Rheological Properties of Buffalo Yoghurt Samples

The physicochemical and rheological properties of yoghurts during the storage period are presented in Table 1. At the beginning of storage, the lowest pH value was measured in Group 2 (4.77 ± 0.01), while the highest was observed in Group 1 (5.35 ± 0.04). The pH values of all groups decreased over the 28‐day storage period. Compared to the control, the pH values of the groups containing *L. acidophilus* and *B. lactis* strains at the end of storage were similar and higher. However, at the end of storage, Group 2, with the lowest pH value of 4.39 ± 0.03, showed that using *Lpb. plantarum* Lb9 in yoghurt may improve acidity values. The highest titratable acidity was in Group 2 at the beginning and end of storage, consistent with the pH results. Titratable acidity values increased until Day 14 of storage, but on Day 28, the percentage acidity values of all samples were similar to those at the beginning of storage. Differences in storage time and groups had no statistically significant effect on dry matter and fat contents (*p* ˂ 0.05). Furthermore, the protein contents of all samples at all storage stages were statistically similar. Rheological results of the control and probiotic addition buffalo yoghurt samples indicated that the viscosity of all samples was directly related to the storage period (*p* < 0.05). The control group exhibited a slower increase in viscosity due to a slightly slower decrease in pH. Group 2 samples had higher viscosity values than other probiotic groups on the 28th day of storage.

**TABLE 1:** Physicochemical and rheological properties of yoghurts during cold storage.

		Storage (day)		
	Yoghurt sample	Day 1	Day 14	Day 28
pH	Control	5.01 ± 0.01^bA^	4.63 ± 0.02^bB^	4.65 ± 0.02^bB^
	Group 1	5.35 ± 0.04^aA^	4.9 ± 0.01^aB^	4.83 ± 0.02^aB^
	Group 2	4.77 ± 0.01^cA^	4.31 ± 0.01^cB^	4.39 ± 0.03^cB^
	Group 3	5.26 ± 0.02^aA^	4.85 ± 0.01^aB^	4.82 ± 0.03^aB^
Titratable acidity (%)	Control	1.18 ± 0.01^bB^	1.4 ± 0.07^bA^	1.15 ± 0.01^bB^
	Group 1	1.07 ± 0.01^cB^	1.22 ± 0.04^bcA^	0.99 ± 0.02^bB^
	Group 2	1.35 ± 0.01^aB^	1.63 ± 0.05^aA^	1.40 ± 0.07^aB^
	Group 3	1.01 ± 0.01^dB^	1.13 ± 0.03^cA^	1.03 ± 0.03^bAB^
Dry matter (%)	Control	14.19 ± 0.18^aA^	14.75 ± 0.06^aA^	14.46 ± 0.15^aA^
	Group 1	13.88 ± 0.03^aA^	14.6 ± 0.11^aA^	14.2 ± 0.34^aA^
	Group 2	13.98 ± 0.08^aA^	14.37 ± 0.16^aA^	14.29 ± 0.12^aA^
	Group 3	14.01 ± 0.10^aA^	14.41 ± 0.14^aA^	14.23 ± 0.07^aA^
Fat (%)	Control	4.80 ± 0.00^aA^	4.6 ± 0.07^aA^	4.7 ± 0.07^aA^
	Group 1	4.80 ± 0.14^aA^	4.8 ± 0.00^aA^	4.8 ± 0.14^aA^
	Group 2	4.90 ± 0.07^aA^	4.7 ± 0.14^aA^	4.9 ± 0.00^aA^
	Group 3	4.70 ± 0.07^aA^	4.9 ± 0.14^aA^	4.9 ± 0.14^aA^
Protein (%)	Control	5.12 ± 0.16^aA^	5.37 ± 0.07^aA^	5.45 ± 0.01^aA^
	Group 1	5.14 ± 0.04^aB^	5.34 ± 0.02^aA^	5.37 ± 0.01^aA^
	Group 2	5.16 ± 0.02^aB^	5.33 ± 0.02^aA^	5.38 ± 0.01^aA^
	Group 3	5.33 ± 0.02^aB^	5.50 ± 0.01^aA^	5.42 ± 0.03^aAB^
Apparent viscosity (cp)	Control	1.77 ± 0.58^bA^	1.82 ± 0.11^bA^	1.86 ± 0.21^bA^
	Group 1	2.25 ± 0.26^abA^	2.34 ± 0.50^abA^	2.41 ± 0.47^abA^
	Group 2	2.87 ± 0.38^aA^	2.89 ± 0.44^aA^	2.99 ± 0.51^aA^
	Group 3	2.13 ± 0.14^abA^	2.16 ± 0.13^abA^	2.38 ± 0.24^abA^

*Note*: a, b, c indicate the difference between averages with different letters in the same column (*p* < 0.05). A, B, C indicate the difference between averages with different letters on the same line (*p* < 0.05).

### 3.2 Viability of Probiotics and Other Microorganisms in Buffalo Yoghurt Samples During the Fermentation

**FIGURE 2:**
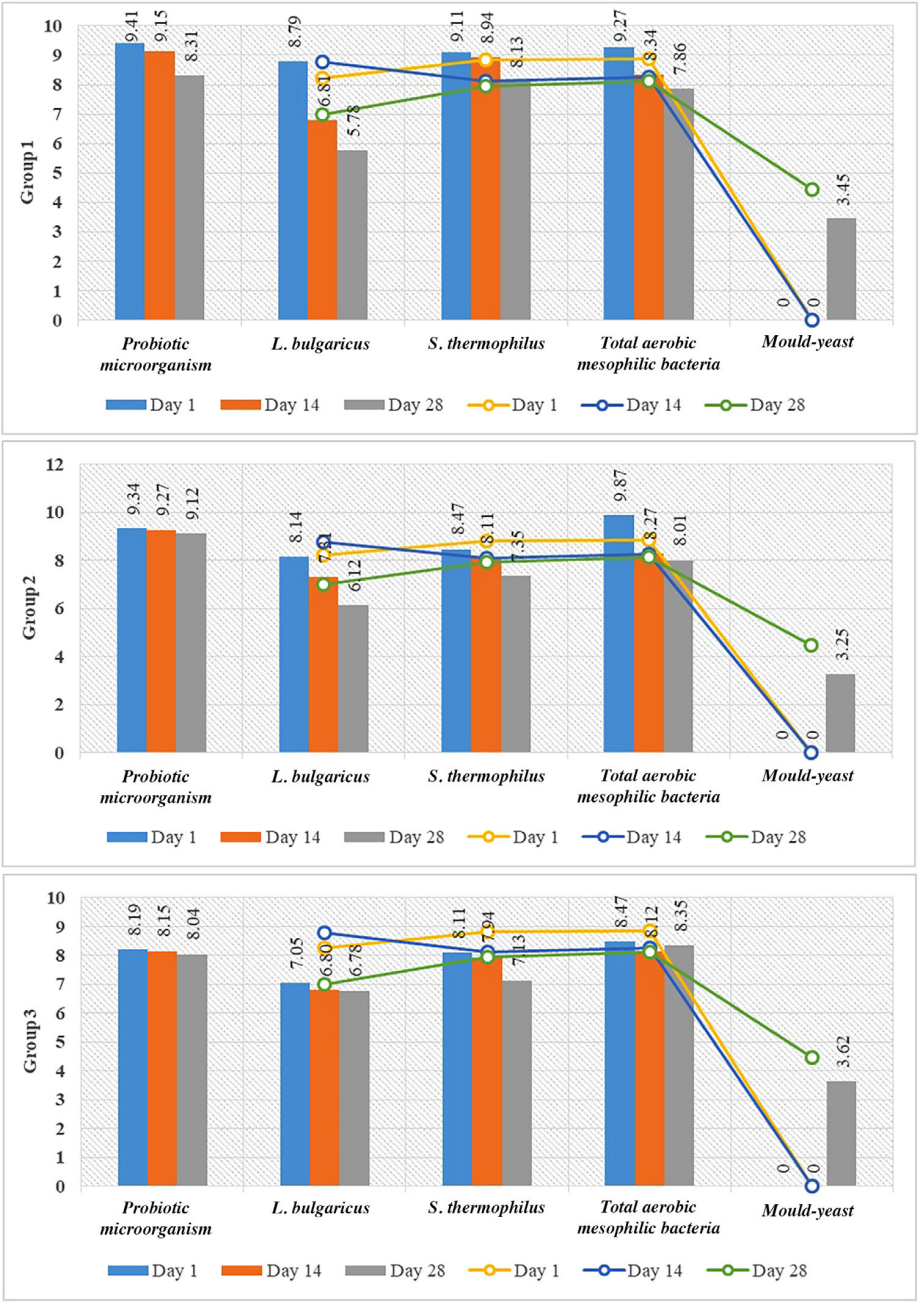
The comparison of the viability assessment of probiotics and other microorganisms in buffalo yoghurt groups (Groups 1–3) during the storage period, relative to the control group.

The comparison of the viability assessment of probiotics and other microorganisms in yoghurt samples during the storage period, relative to the control group, is presented in Figure 2. Adding probiotics at different rates to the trial buffalo yoghurt samples and the storage period had a statistically significant effect (*p* < 0.05) on the number of *Streptococcus thermophilus*, *Lactobacillus bulgaricus* and probiotic microorganisms. The initial *S. thermophilus* count, which was 9.11, 8.48 and 8.11 log CFU/g in Groups 1–3, respectively, decreased by approximately 1 logarithm by the end of the analysis. The highest *S. thermophilus* numbers were detected in the group to which *L. acidophilus* was added. The *L. bulgaricus* count in Group 1 was 5.78 log CFU/g by the end of 28 days. As shown in Figure 2, the initial count of viable cells in all probiotic groups ranged from 8 to 9 log CFU/g. After a 28‐day fermentation period, there was a decrease of approximately 1 logarithm in all probiotic groups. The average viable cell count for all groups at the end of this period was approximately 8 logarithms. For the groups, including *B. lactis* and *L. acidophilus* strains, the viability was 8.04 and 8.31 log CFU/g by the end of the analysis, respectively. Group 2, including the *Lpb. plantarum* strain, demonstrated a minor reduction in viability from 9.27 to 9.12 log CFU/g between 14 and 28 days. In this study, the average mould yeast count for all probiotic groups reached 3 log CFU/g at the end of fermentation.

### 3.3 Peptide Profiles of WSEs by RP‐HPLC

**FIGURE 3:**
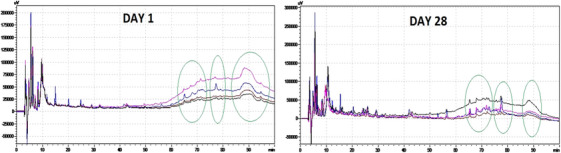
RP‐HPLC peptide profiles of WSEs of buffalo yoghurt samples. Black‐coloured peak: control; Group 1: pink‐coloured peak; Group 2: blue‐coloured peak; Group 3: red‐coloured peak.

Figure 3 presents the RP‐HPLC chromatogram images of WSE obtained at 1 and 28 days of storage for the yoghurts produced in this study. In all yoghurt samples, the peak heights of hydrophobic peptides decreased by the 28th day of storage. A notable reduction in peak heights is observed after the 60th min of the chromatogram, as highlighted by the green circle. Despite a high degree of similarity in the number of peaks and retention times among the groups in this region, it was found that peak heights varied according to the culture used, both at the beginning and at the end of the storage period. While Group 1 exhibited the highest peak height at the start of storage, the peak heights of yoghurts containing probiotics were comparable at the end of storage. The control group, which initially showed low peak heights, exhibited the highest at the end of storage. This observation could be attributed to the higher proteolytic activity of probiotic cultures during fermentation compared to commercial yoghurt cultures.

### 3.4 ACE‐Inhibitor and Antioxidant Activity of WSEs of Buffalo Yoghurt Samples

This study presents the ACE‐inhibitory and antioxidant activity results of WSEs from buffalo yoghurt samples, as detailed in Table 2. The ACE‐inhibitory effect of WSEs from all buffalo yoghurts increased over the 28‐day storage period. Initially, the ACE‐inhibitory effect of WSEs from probiotic cultures was significantly higher than the control sample (*p* < 0.05). Specifically, at the start of storage, Group 1, containing *L. acidophilus*, exhibited the highest ACE‐inhibitory activity at 19.60% (*p* < 0.05). By the end of the storage period, all samples demonstrated statistically similar ACE‐inhibitory activities (*p* > 0.05). These results underscore the importance of using probiotic cultures in addition to yoghurt cultures to enhance ACE‐inhibitory peptide release at the beginning of storage.

**TABLE 2:** Bioactive properties of WSEs of yoghurt samples during cold storage.

	Yoghurt samples	Day 1	Day 28
ACE‐inhibitory activity (%)	Control	8.27 ± 0.38^cB^	21.63 ± 0.51^aA^
	Group 1	19.6 ± 0.50^aA^	23.54 ± 0.9^aA^
	Group 2	10.81 ± 0.64^cB^	21.37 ± 0.51^aA^
	Group 3	14.89 ± 0.64^bB^	22.26 ± 0.90^aA^
Antioxidant activity (%)	Control	21.14 ± 2.99^bB^	44.12 ± 1.80^bA^
	Group 1	20.3 ± 1.42^bB^	56.45 ± 1.04^bA^
	Group 2	27.75 ± 0.37^abB^	61.48 ± 0.38^aA^
	Group 3	36.2 ± 0.73^aB^	60.34 ± 0.95^aA^

*Note*: a, b, c indicate the difference between averages with different letters in the same column (*p* < 0.05). A, B, C indicate the difference between averages with different letters on the same line (*p* < 0.05).

As shown in Table 2, the antioxidant activity of all samples increased during storage. At the end of the storage period, Groups 2 and 3 demonstrated the highest antioxidant activity, with values of 61.48% and 60.34%, respectively (*p* < 0.05). At the end of storage, the control group showed the lowest antioxidant activity. These results show that the addition of *Lpb. plantarum* and *B. lactis* significantly increased the antioxidant activity in buffalo yoghurt at the end of storage.

### 3.5 ACE‐Inhibitor and Antioxidant Activity of Buffalo Yoghurt Samples With Gastrointestinal Digestion

The ACE‐inhibitory and antioxidant activity of digested buffalo yoghurt samples are detailed in Table 3. The table illustrates that the ACE inhibition effects of peptides released in buffalo milk due to probiotic addition differed from those observed after gastrointestinal digestion. This variation is attributed to the proteolytic activity of digestive enzymes on peptides and the differing ability of newly released peptides to bind to ACE. Following gastric digestion, yoghurts produced with control and Group 3 exhibited the highest ACE‐inhibitory effects at the beginning of storage. However, yoghurts produced with Groups 2 and 3 by the end of storage showed the highest ACE‐inhibitory effects. After intestinal digestion, both at the beginning and end of storage, the OUT phase demonstrated the highest ACE‐inhibitory activity across all yoghurt samples (*p* < 0.05), which was an increase compared to undigested samples. The ACE inhibition in IN phase samples was higher at the beginning of storage compared to the gastric‐digested samples, except for the control group.

**TABLE 3:** ACE‐inhibitory and antioxidant activity (%) of digestion examples of yoghurts during cold storage.

			Digestion examples of yoghurts				
	Yoghurt samples	Day 1			Day 28		
		AD	OUT	IN	AD	OUT	IN
**ACE‐inhibitory activity (%)**	Control	19.59 ± 0.40^Ba^	28.80 ± 0.23^Aab^	15.78 ± 0.35^Cc^	10.39 ± 0.56^Yy^	24.86 ± 0.70^Xz^	25.80 ± 0.56^Xx^
	Group 1	17.28 ± 0.50^Ba^	29.95 ± 1.15^Aa^	25.69 ± 0.60^Aa^	21.91 ± 0.84^Yx^	32.02 ± 0.56^Xx^	15.31 ± 0.42^Zz^
	Group 2	16.71 ± 1.04^Ba^	29.61 ± 1.27^Aa^	20.16 ± 1.04^Bbc^	24.30 ± 0.70^Xx^	27.30 ± 0.56^Xyz^	16.90 ± 0.56^Yz^
	Group 3	20.51 ± 0.70^Aa^	22.12 ± 1.84^Ab^	23.04 ± 0.92^Aab^	25.14 ± 0.42^XYx^	28.23 ± 0.42^Xy^	22.19 ± 0.84^Yy^
**Antioxidant activity (%)**	Control	24.40 ± 0.92^Aa^	15.74 ± 0.64^Ba^	10.06 ± 0.33^Cb^	25.64 ± 0.78^Xx^	13.30 ± 0.59^Yx^	9.30 ± 0.68^Zx^
	Group 1	23.11 ± 0.44^Aa^	17.43 ± 0.92^Ba^	12.85 ± 0.54^Ca^	27.20 ± 0.39^Xx^	14.58 ± 0.29^Yx^	10.67 ± 0.88^Zx^
	Group 2	23.71 ± 0.62^Aa^	11.75 ± 0.63^Bb^	10.46 ± 0.52^Bb^	24.17 ± 0.68^Xx^	15.85 ± 0.39^Yx^	9.69 ± 0.40^Zx^
	Group 3	25.60 ± 0.71^Aa^	16.04 ± 0.54^Ba^	9.46 ± 0.32^Cb^	25.73 ± 0.29^Xx^	16.63 ± 1.17^Yx^	11.64 ± 0.68^Zx^

*Note*: a, b, c; x, y, z indicate the difference between averages with different letters in the same column (*p* < 0.05). A, B, C; X, Y, Z indicate the difference between averages with different letters on the same line (*p* < 0.05).

As shown in Table 3, storage reduced antioxidant activity for all yoghurt samples during gastric and intestinal digestion compared to undigested samples. Among the digestion phases, the highest antioxidant activity for all yoghurt samples during storage was observed after gastric digestion (*p* < 0.05). However, antioxidant activities for all samples subjected to gastric digestion were statistically similar (*p* > 0.05). A decrease in antioxidant activity was noted after intestinal digestion, with the lowest activity recorded in the IN phase (*p* < 0.05). On the first day of storage, Group 1's IN digestion phase exhibited the highest antioxidant activity at 12.85%, significantly higher than other yoghurt samples (*p* < 0.05). No significant differences were observed among the samples at the end of storage (*p* > 0.05). In Group 2, the antioxidant activity of samples increased by the end of storage after intestinal digestion. Nonetheless, at the end of storage, all samples from the intestinal digestion phases were comparable to the control group.

### 3.6 Cholesterol Levels of Buffalo Yoghurts

**FIGURE 4:**
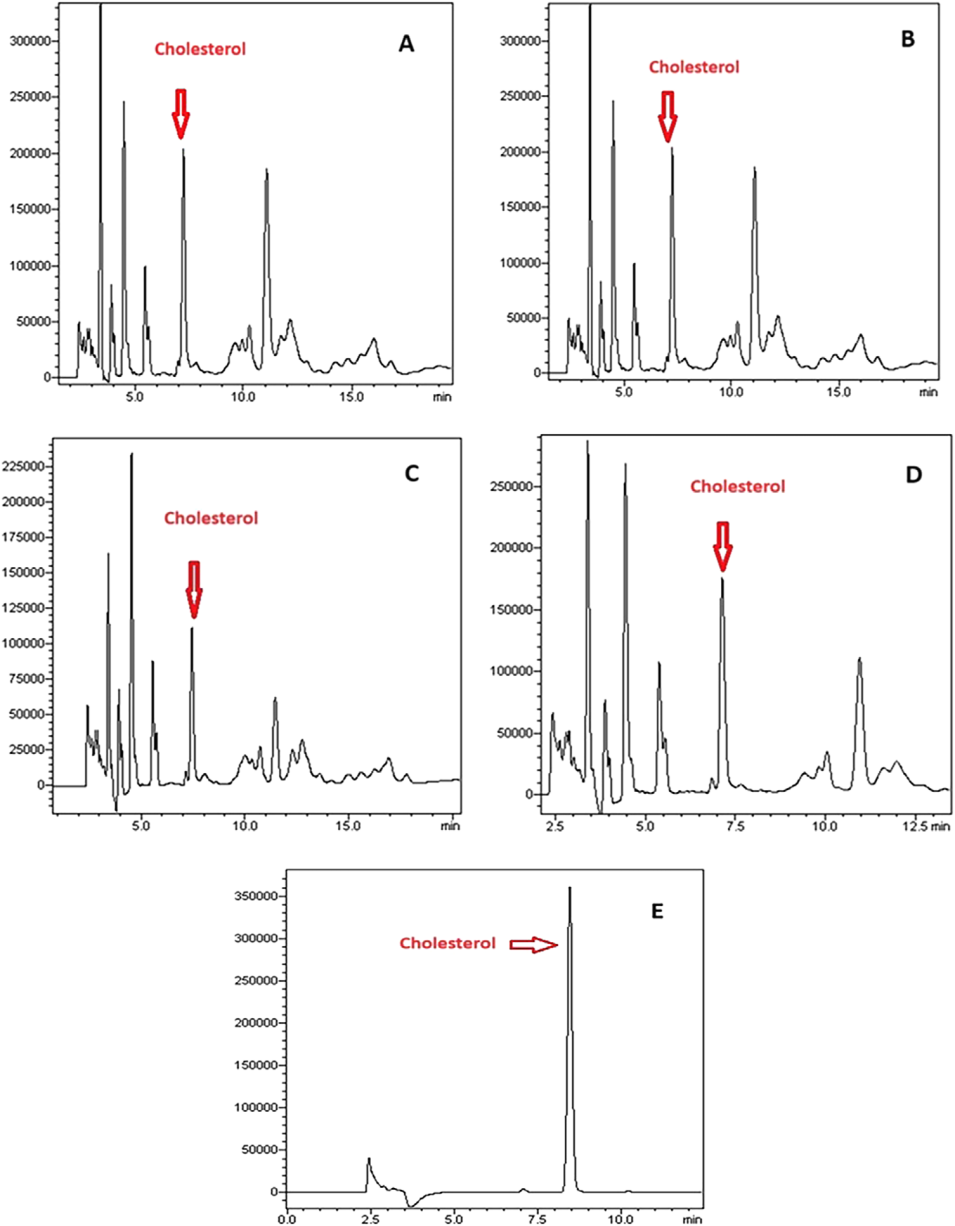
Cholesterol chromatograms determined by HPLC in buffalo yoghurt samples. A peak: control group chromatogram on the 28th day; B peak: Group 1 chromatogram on the 28th day; C peak: Group 2 chromatogram on the 28th day; D peak: Group 3 chromatogram on the 28th day; E peak: chromatogram of cholesterol standard.

Buffalo yoghurt samples were analysed for their cholesterol‐reducing properties, and the cholesterol chromatograms obtained via HPLC are presented in Figure 4. The chromatogram for the cholesterol standard, labelled with code E in Figure 4, reveals a prominent peak representing cholesterol between the 5th and 10th min. This peak was consistently observed in the chromatograms of all yoghurt samples within this time frame, indicating the presence of cholesterol. Among the yoghurt samples, those produced with *Lpb. plantarum* exhibited the lowest cholesterol levels. This was followed by yoghurt produced with the *B. lactis* strain.

## 4 Discussion

Acidity values vary due to bacteria releasing lactic acid from lactose metabolism during storage. The release of lactic acid during storage leads to a decrease in pH values and an increase in titratable acidity. Previous studies have reported that the pH value in yoghurts decreases during storage, which is consistent with our results (Akgun et al. 2016). It has been reported that the use of *Lpb. plantarum* WCFS1 with traditional cultures increases lactic acid yield (Zhang et al. 2020). Another study found that using *Bifidobacterium* strains reduced yoghurt acidity (Turgut and Cakmakci 2018). Similar to our study, many researchers have reported that yoghurts' dry matter and fat contents do not change during storage (Junaid et al. 2023; Younas et al. 2024).

The apparent viscosity of the probiotic addition groups increased at the end of the storage period. This study's results agree with the findings of Akpınar et al. (2020), who also observed a relationship between the viscosity of yoghurt samples and the storage period. Moreover, this phenomenon might be attributed to a decline in the acidity of the samples as the storage period increased. The pH of yoghurts induces a reduction in the electronegativity of casein micelles, consequently diminishing the repulsive forces between casein molecules, as noted by Lee and Lucey (2010). Furthermore, the strains of LAB can produce exopolysaccharides (EPS) during fermentation and gel formation, thereby enhancing the texture of yoghurt (Dikmen et al. 2024). Similarly, within the scope of this study, yoghurt produced with *Lpb. plantarum* had higher viscosity values at the end of the storage period. *Lpb. plantarum* may have produced a higher level of EPS than other probiotics.

*L. bulgaricus* and *S. thermophilus* play pivotal roles in the fermentation process of yoghurt, contributing to its distinctive taste and aroma and fundamentally determining its sensory attributes (Terpou et al. 2019; Ayivi and İbrahim 2022). *L. bulgaricus* possesses multiple incomplete carbohydrate metabolic pathways and exhibits a predilection for growth in lactose‐rich environments, facilitating efficient metabolic processes. The strain is characterized by a robust proteolytic system and an efficient amino acid transport mechanism, augmenting its metabolic capabilities, especially in environments rich in protein, such as milk and yoghurt (Canon et al. 2020). The growth of *L. bulgaricus* was supported in buffalo yoghurts, to which different probiotic strains were added. The microbial count consistently remained at 6 logarithms or higher for the other probiotic groups. Similar findings were reported for buffalo yoghurt with probiotic addition (Terzioğlu et al. 2023). Group 2 had the highest survivability of probiotic microorganisms at the end of the storage period. This outcome might be attributed to *Lpb. plantarum* potentially exhibiting a rapid response to stress factors, including low pH. Moreover, the *Lpb. plantarum* strain, isolated from cheese and previously identified to possess cholesterol assimilation and bile salt hydrolase (BSH) activity properties, decreased by approximately 1 logarithm by the end of the storage, maintaining the highest count in buffalo yoghurt until the end of the 28 days.

It was determined that the number of probiotic microorganisms in the trial yoghurt samples during the storage period was higher than 10^6^ CFU/g, the probiotic product limit to benefit from the therapeutic effect. Present research results agree with studies examining probiotic microorganisms' development (Hamdy et al. 2021; Vargas‐Ramella et al. 2021). Throughout storage, the LAB within the product exhibits growth and metabolic activity, breaking down carbohydrates into organic acids. The accumulation of these acids leads to a decrease in pH. The LAB contributes to increased aroma components, influencing the final product's technological properties and microbial stability (Fırıncıoğulları and Öner 2022). In light of the present study, the selected probiotic *Lpb. plantarum* strain proves effective in buffalo yoghurts, serving as a suitable substrate and significantly enhancing the product's quality. Yeast metabolism entails the conversion of carbohydrates into ethanol, carbon dioxide (CO_2_) and various secondary products, playing a crucial role in alcohol fermentation. However, mould growth is undesirable in a healthy yoghurt fermentation process. Findings obtained in the light of this study are generally consistent with the mould‐yeast count ranges reported by Yalçın and Polat (2023).

It has been reported in the literature that the addition of probiotics increases the proteolysis of casein and as a result, leads to the formation of a greater number of peaks in the chromatogram profile (Pinto et al. 2020). Similarly, in this study, it was observed that probiotic cultures caused different peak profiles during storage. While peptide release was higher at the beginning of storage in the groups containing probiotics compared to the control group, the peptides formed by the end of storage may have been degraded, which could explain the lower amount compared to the control group. In dairy products, the ACE‐inhibitory activity of peptides generated through fermentation is influenced by various factors, including the type of milk used, the pre‐processing applied to the milk and the composition of the starter culture. The addition of probiotic cultures to dairy products enhances peptide formation more effectively than commercially used starter cultures, with the level of peptides released varying according to the stage of fermentation (Zhou et al. 2019; Khakhariya et al. 2023; Uğur and Öner 2023). Previous studies have indicated that yoghurt may be a natural source of ACE inhibitors (Terzioğlu et al. 2023). Kim et al. (2021) investigated the ACE‐inhibitory effects of yoghurts produced with *L. rhamnosus* GG KCTC 12202 BP, *Lpb. plantarum* KU15003 (T2), *Lpb. plantarum* KU15031, *Lpb. plantarum* NK181 and *L. bulgaricus* KU200171 cultures, finding that all strains exhibited higher ACE‐inhibitory effects compared to the control group. Erkaya‐Kotan (2020) also reported increased ACE‐inhibitor activity in probiotic yoghurts during storage.

Bioactive compounds in fermented dairy products are crucial in mitigating the effects of reactive oxygen species, including superoxide, hydroxyl and peroxyl radicals. These compounds, which encompass peptides, free amino acids, enzymes and other substances, are essential for maintaining redox balance in living organisms (Gjorgievski et al. 2014; Ayyash et al. 2018). The production of antioxidant peptides and the development of radical scavenging activity are strain‐specific traits linked to proteolysis (Sah et al. 2016). Aloğlu and Öner (2011) reported an enhancement in the antioxidant activity of commercial yoghurt WSEs after 4 weeks of storage. Similarly, Taha et al. (2017) found that antioxidant activity increased at the end of storage in buffalo yoghurt produced with various probiotic cultures, with WSEs of buffalo yoghurt fermented with *L. helveticus* CH5 exhibiting the highest antioxidant activity compared to yoghurt culture and *L. acidophilus* 20552. Consistent with these findings, other studies have reported increased antioxidant activity during storage in yoghurts produced with different probiotic strains (Erkaya‐Kotan 2020; Kim et al. 2021).

Previous studies have indicated that yoghurt may be a natural source of ACE inhibitors (Terzioğlu et al. 2023). Nguyen et al. (2020) found that peptides released during gastrointestinal digestion, particularly antihypertensive peptides, were more prevalent in yoghurt samples than in milk. Similarly, Jin et al. (2016) reported that ACE‐inhibitory peptides released through gastric and pancreatic digestion in yoghurt increased ACE‐inhibitory activity. Research on the antioxidant activity of yoghurt post‐gastrointestinal digestion has yielded varied results. Diep et al. (2022) reported that yoghurt with varying amounts of tortilla powder exhibited increased antioxidant activities following digestion. In contrast, Anuyahong et al. (2020) found that yoghurt enriched with rice fruit extract showed an initial increase in antioxidant activity up to 120 min post‐gastric digestion, followed by a decrease. In this study, antioxidant activity showed differences compared to undigested yoghurt samples. These variations in antioxidant activity can be attributed to the breakdown of milk proteins into peptides and amino acids by LAB and proteolytic enzymes in the stomach (Sah et al. 2014; Akbal and Öner 2021).

In this study, the control group not including probiotics had the highest cholesterol level. Fırıncıoğulları and Öner (2022) reported that a decrease in cholesterol content was observed in fermented products produced using *Lpb. plantarum* and *Lpb. paracasei* compared to the control group. In this study, it was observed that the group added *Lpb. plantarum* strain, which has high cholesterol assimilation properties, showed the highest assimilation compared to other yoghurt samples, and this situation coincides with the study conducted by Fırıncıoğulları and Öner (2022). In light of all the findings, the *Lpb. plantarum* strain used with buffalo yoghurt as a probiotic culture could be an effective strategy to overcome hypercholesterolaemia due to its high cholesterol assimilation properties.

## 5 Conclusion

The present study examined the efficacy of utilizing various LAB cultures with high cholesterol assimilation capabilities and probiotic properties in producing buffalo yoghurt with enhanced physicochemical, microbiological and bioactive attributes. The findings indicated that yoghurt produced with *Lpb. plantarum* exhibited superior viability of probiotic microorganisms and higher cholesterol assimilation rates by the end of storage than the control group. The incorporation of probiotic cultures significantly enhanced the release of ACE‐inhibitory peptides, with both antioxidant and ACE‐inhibitory activities increasing throughout the storage period in all samples. Optimizing fermented dairy products with diverse culture combinations or augmenting their functionality with additional probiotics may offer prolonged therapeutic benefits. Buffalo yoghurt incorporating these cultures is anticipated to emerge as an innovative product among alternative dairy options. Nonetheless, further in vivo studies are required to elucidate these microorganisms' hypocholesterolaemic and functional effects.

## Author Contributions

**Sinan Akbal**: conceptualization, methodology, software. **Esra Uğur Geçer**: conceptualization, methodology, software. **Pelin Ertürkmen**: conceptualization, methodology.

## Ethics Statement

The authors have nothing to report.

## Conflicts of Interest

The authors declare no conflicts of interest.

### Peer Review

The peer review history for this article is available at https://www.webofscience.com/api/gateway/wos/peer‐review/10.1002/vms3.70233.

## Data Availability

The data that support the findings of this study are available from the corresponding author upon reasonable request.

## References

[vms370233-bib-0001] Akbal, S. , and Z. Öner . 2021. “Süt Ve Süt Ürünlerinden Elde Edilen Peptitlerin Patojen Mikroorganizmalar Üzerine Antimikrobiyal Etkisi.” Kırklareli Üniversitesi Mühendislik Ve Fen Bilimleri Dergisi 7, no. 2: 305–322.

[vms370233-bib-0002] Akgun, A. , F. Yazici , and H. A. Gulec . 2016. “Effect of Reduced fat content on the Physicochemical and Microbiological Properties of Buffalo Milk Yoghurt.” LWT 74: 521–527.

[vms370233-bib-0003] Akpınar, A. , D. Saygılı , and O. Yerlikaya . 2020. “Production of Set‐Type Yoghurt Using *Enterococcus Faecium* and *Enterococcus Durans* Strains With Probiotic Potential as Starter Adjuncts.” International Journal of Dairy Technology 73, no. 4: 726–736.

[vms370233-bib-0004] Albuquerque, T. G. , M. B. P. P. Oliveira , A. Sanches‐Silva , and H. S. Costa . 2016. “Cholesterol Determination in Foods: Comparison Between High Performance and Ultra‐High Performance Liquid Chromatography.” Food Chemistry 193: 18–25. 26433282 10.1016/j.foodchem.2014.09.109

[vms370233-bib-0005] Aloğlu, H. Ş. , and Z. Öner . 2011. “Determination of Antioxidant Activity of Bioactive Peptide Fractions Obtained From Yoghurt.” Journal of Dairy Science 94, no. 11: 5305–5314. 22032353 10.3168/jds.2011-4285

[vms370233-bib-0006] Al‐Shaikh, S. A. H. , M. A. Alhamid , and A. A. Aldhalemi . 2020. “Study of the Effect of Fortified Milk by Zinc Salts in Different Concentrations on the Sensory and Physiochemical Properties of the Processed Yoghurt.” Plant Archives 20, no. 2: 81–90.

[vms370233-bib-0007] Aluko, R. E. , and E. Monu . 2003. “Functional and Bioactive Properties of Quinoa Seed Protein Hydrolysates.” Journal of Food Science 68, no. 4: 1254–1258.

[vms370233-bib-0008] Anuyahong, T. , C. Chusak , and S. Adisakwattana . 2020. “Incorporation of Anthocyanin‐Rich Riceberry Rice in Yoghurts: Effect on Physicochemical Properties, Antioxidant Activity and in Vitro Gastrointestinal Digestion.” LWT 129: 109571.

[vms370233-bib-0009] AOAC . 1990. Official Methods of Analysis. 15th ed. Association of Official Analytical Chemists.

[vms370233-bib-0010] Asan‐Ozusaglam, M. , and A. Gunyakti . 2019. “ *Lactobacillus fermentum* Strains From Human Breast Milk With Probiotic Properties and Cholesterol‐Lowering Effects.” Food Science and Biotechnology 28: 501–509. 30956862 10.1007/s10068-018-0494-y PMC6431312

[vms370233-bib-0011] Ayivi, R. D. , and S. A. Ibrahim . 2022. “Lactic Acid Bacteria: An Essential Probiotic and Starter Culture for the Production of Yoghurt.” International Journal of Food Science & Technology 57, no. 11: 7008–7025.

[vms370233-bib-0012] Ayyash, M. , A. K. Al‐Nuaimi , S. Al‐Mahadin , and S. Q. Liu . 2018. “In Vitro Investigation of Anticancer and ACE‐Inhibiting Activity, α‐Amylase and α‐Glucosidase Inhibition, and Antioxidant Activity of Camel Milk Fermented With Camel Milk Probiotic: A Comparative Study With Fermented Bovine Milk.” Food Chemistry 239: 588–597. 28873609 10.1016/j.foodchem.2017.06.149

[vms370233-bib-0013] Baltova, K. , and Z. Dimitrov . 2014. “Probiotic and Cultural Characteristic of Strain *Lactobacillus gasseri* 4/13 of Human Origin.” Biotechnology & Biotechnological Equipment 28, no. 6: 1084–1088. 26692783 10.1080/13102818.2014.974303 PMC4648341

[vms370233-bib-0014] Basilicata, M. G. , G. Pepe , E. Sommella , et al. 2018. “Peptidome Profiles and Bioactivity Elucidation of Buffalo‐Milk Dairy Products After Gastrointestinal Digestion.” Food Research International 105: 1003–1010. 29433190 10.1016/j.foodres.2017.12.038

[vms370233-bib-0015] Canon, F. , T. Nidelet , E. Guédon , A. Thierry , and V. Gagnaire . 2020. “Understanding the Mechanisms of Positive Microbial Interactions that Benefit Lactic Acid Bacteria Co‐Cultures.” Frontiers in Microbiology 11: 2088. 33013761 10.3389/fmicb.2020.02088 PMC7500094

[vms370233-bib-0016] Chailangka, A. , N. Leksawasdi , W. Ruksiriwanich , et al. 2023. “Natural Ingredients and Probiotics for Lowering Cholesterol and Saturated Fat in Dairy Products: An Updated Review.” Quality Assurance and Safety of Crops and Foods 15, no. 2: 140–160.

[vms370233-bib-0017] Cushman, D. W. , and H. S. Cheung . 1971. “Spectrophotometric Assay and Properties of the Angiotensin‐Converting Enzyme of Rabbit Lung.” Biochemical Pharmacology 20, no. 7: 1637–1648. 4355305 10.1016/0006-2952(71)90292-9

[vms370233-bib-0018] Dave, R. I. , and N. P. Shah . 1998. “Ingredient Supplementation Effects on Viability of Probiotic Bacteria in Yoghurt.” Journal of Dairy Science 81, no. 11: 2804–2816. 9839222 10.3168/jds.S0022-0302(98)75839-4

[vms370233-bib-0019] Derakhshan, M. , S. O. Ghasemian , and M. Gholami‐Ahangaran . 2023. “The Effects of Probiotic and Phytase on Growth Performance, Biochemical Parameters and Antioxidant Capacity in Broiler Chickens.” Veterinary Medicine and Science 9, no. 2: 860–866. 36669151 10.1002/vms3.1075 PMC10029878

[vms370233-bib-0020] Diep, T. T. , M. J. Y. Yoo , and E. Rush . 2022. “Effect of in Vitro Gastrointestinal Digestion on Amino Acids, Polyphenols and Antioxidant Capacity of Tamarillo Yoghurts.” International Journal of Molecular Sciences 23, no. 5: 2526. 35269670 10.3390/ijms23052526 PMC8910476

[vms370233-bib-0021] Dikmen, H. , H. Goktas , F. Demirbas , et al. 2024. “Multilocus Sequence Typing of *L. bulgaricus* and *S. thermophilus* Strains From Turkish Traditional Yoghurts and Characterization of their Techno‐Functional Roles.” Food Science and Biotechnology 33, no. 3: 625–635. 38274192 10.1007/s10068-023-01366-2 PMC10805743

[vms370233-bib-0022] Erkaya‐Kotan, T. 2020. “In vitro Angiotensin Converting Enzyme (ACE)‐Inhibitory and Antioxidant Activity of Probiotic Yoghurt Incorporated With Orange Fibre During Storage.” Journal of Food Science and Technology 57: 2343–2353. 32431360 10.1007/s13197-020-04272-1 PMC7230084

[vms370233-bib-0067] Ertürkmen, P. , B. Fırıncıoğulları , and Z. Öner . 2023. “The Expression Levels of Genes Responsible for the Enzymatic Activity of Bile Salt Hydrolase (BSH) and the Relationship of Cholesterol Assimilation in *L. plantarum* and *L. paracasei* .” Current Microbiology 80, no. 6: 205. 37156986 10.1007/s00284-023-03311-2

[vms370233-bib-0023] Farzamirad, V. , and R. E. Aluko . 2008. “Angiotensin‐Converting Enzyme Inhibition and Free‐Radical Scavenging Properties of Cationic Peptides Derived From Soybean Protein Hydrolysates.” International Journal of Food Sciences and Nutrition 59, no. 5: 428–437. 18636366 10.1080/09637480701592897

[vms370233-bib-0024] Fırıncıoğulları, B. , and Z. Öner . 2022. “Kolesterol Düşürücü Etkilere Sahip *Lactobacillus* spp. Suşlarının Peynirde Başlatıcı Kültür Olarak Kullanımı.” Gıda 47, no. 2: 266–276.

[vms370233-bib-0025] Gjorgievski, N. , J. Tomovska , G. Dimitrovska , B. Makarijoski , and M. A. Shariati . 2014. “Determination of the Antioxidant Activity in Yoghurt.” Journal of Hygienic Engineering and Design 8: 88–92.

[vms370233-bib-0026] Górska, A. , D. Przystupski , M. J. Niemczura , and J. Kulbacka . 2019. “Probiotic Bacteria: A Promising Tool in Cancer Prevention and Therapy.” Current Microbiology 76: 939–949. 30949803 10.1007/s00284-019-01679-8 PMC6586914

[vms370233-bib-0027] Hamdy, S. M. , H. S. Abdelmontaleb , A. M. Mabrouk , and K. A. Abbas . 2021. “Physicochemical, Viability, Microstructure, and Sensory Properties of Whole and Skimmed Buffalo Set‐Yoghurts Containing Different Levels of Polydextrose During Refrigerated Storage.” Journal of Food Processing and Preservation 45, no. 7: e15643.

[vms370233-bib-0028] Hao, X. , W. Yang , Q. Zhu , et al. 2021. “Proteolysis and ACE‐Inhibitory Peptide Profile of Cheddar cheese: Effect of Digestion Treatment and Different Probiotics.” LWT 145: 111295.

[vms370233-bib-0029] IDF . 1987. Determination of Total Solids Content. IDF Standard 21B. International Dairy.

[vms370233-bib-0030] IDF . 1993. Milk, Determination of Nitrogen Content. FIL‐IDF 20B. International Dairy.

[vms370233-bib-0031] Jin, Y. , Y. Yu , Y. Qi , F. Wang , J. Yan , and H. Zou . 2016. “Peptide Profiling and the Bioactivity Character of Yoghurt in the Simulated Gastrointestinal Digestion.” Journal of Proteomics 141: 24–46. 27108547 10.1016/j.jprot.2016.04.010

[vms370233-bib-0032] Jitpakdee, J. , D. Kantachote , H. Kanzaki , and T. Nitoda . 2021. “Selected Probiotic Lactic Acid Bacteria Isolated From Fermented Foods for Functional Milk Production: Lower Cholesterol With More Beneficial Compounds.” LWT 135: 110061.

[vms370233-bib-0033] Junaid, M. , S. Inayat , N. Gulzar , et al. 2023. “Physical, Chemical, Microbial, and Sensory Evaluation and Fatty Acid Profiling of Value‐Added Drinking Yoghurt (laban) Under Various Storage Conditions.” Journal of Dairy Science 106, no. 1: 39–46. 36357201 10.3168/jds.2022-22358

[vms370233-bib-0034] Karahan, A. G. , B. Arıdoğan‐Cicioğlu , and M. L. Çakmakçı . 2002. “Genel Mikrobiyoloji Uygulama Kılavuzu.” SDÜ 24: 171.

[vms370233-bib-0035] Karakuş, M. Ş. 2013. “Contains Prebiotic Fiber Effect on Some Quality Characteristics Strawberry Flavored of Acidophilus‐Bifidus Yoghurts to the Addition of Stevia.” PhD diss., Harran University.

[vms370233-bib-0036] Khakhariya, R. , A. A. Sakure , R. Maurya , et al. 2023. “A Comparative Study of Fermented Buffalo and Camel Milk With Anti‐Inflammatory, ACE‐Inhibitory and Anti‐Diabetic Properties and Release of Bio Active Peptides With Molecular Interactions: In Vitro, in Silico and Molecular Study.” Food Bioscience 52: 102373. 10.3390/foods12102006 PMC10216992 37238823

[vms370233-bib-0037] Kim, E. D. , H. S. Lee , K. T. Kim , and H. D. Paik . 2021. “Antioxidant and Angiotensin‐Converting Enzyme (ACE) Inhibitory activities of yoghurt supplemented With *Lactiplantibacillus plantarum* NK181 and *Lactobacillus delbrueckii* KU200171 and sensory evaluation.” Foods 10, no. 10: 2324. 34681373 10.3390/foods10102324 PMC8534810

[vms370233-bib-0038] Lee, W. J. , and J. A. Lucey . 2010. “Formation and Physical Properties of Yoghurt.” Journal of Animal Science 23, no. 9: 1127–1136.

[vms370233-bib-0068] McDougall, G. J. , P. Dobson , P. Smith , A. Blake , and D. Stewart . 2005. “Assessing Potential Bioavailability of Raspberry Anthocyanins Using an In Vitro Digestion System.” Journal of Agricultural and Food Chemistry 53, no. 15: 5896–5904. 16028971 10.1021/jf050131p

[vms370233-bib-0039] Meira, S. M. M. , D. J. Daroit , V. E. Helfer , et al. 2012. “Bioactive Peptides in Water‐Soluble Extracts of Ovine Cheeses From Southern Brazil and Uruguay.” Food Research International 48: 322–329.

[vms370233-bib-0040] Munir, M. , M. Nadeem , M. T. Qureshi , et al. 2020. “Effect of Sonication, Microwaves and High‐Pressure Processing on ACE‐Inhibitory Activity and Antioxidant Potential of Cheddar Cheese During ripening.” Ultrasonics Sonochemistry 67: 105140. 32388000 10.1016/j.ultsonch.2020.105140

[vms370233-bib-0041] Nguyen, H. T. , J. L. Gathercole , L. Day , and J. E. Dalziel . 2020. “Differences in Peptide Generation Following in Vitro Gastrointestinal Digestion of Yoghurt and Milk From Cow, Sheep and Goat.” Food Chemistry 317: 126419. 32088406 10.1016/j.foodchem.2020.126419

[vms370233-bib-0042] Nurcahyani, I. , A. Susilowati , and A. Pangastuti . 2023. “Cholesterol‐Lowering Activity by Lactic Acid Bacteria Isolated From Yogurt From Boyolali, Indonesia.” Asian Journal of Natural Product Biochemistry 21, no. 1: 34–45.

[vms370233-bib-0043] Öner, Z , and H. Ş. Aloğlu , eds. 2018. Süt Ve Süt Ürünleri Analiz Yöntemleri. SIDAS.

[vms370233-bib-0044] Öner, Z. , and A. M. Sarıdağ . 2019. “Keçi Sütünden Üretilmiş Beyaz Peynirlerde Olgunlaşma Süresince Meydana Gelen Değişimler.” Gıda 44, no. 3: 523–533.

[vms370233-bib-0045] Palaniyandi, S. A. , K. Damodharan , J. W. Suh , and S. H. Yang . 2020. “Probiotic Characterization of Cholesterol‐Lowering *Lactobacillus Fermentum* MJM60397.” Probiotics and Antimicrobial Proteins 12: 1161–1172. 31432401 10.1007/s12602-019-09585-y

[vms370233-bib-0046] Pinto, G. , G. Picariello , F. Addeo , L. Chianese , A. Scaloni , and S. Caira . 2020. “Proteolysis and Process‐Induced Modifications in Synbiotic Yogurt Investigated by Peptidomics and Phosphopeptidomics.” Journal of Agricultural and Food Chemistry 68, no. 32: 8744–8754. 32678598 10.1021/acs.jafc.0c02603

[vms370233-bib-0047] Ranasinghe, J. G. S. , and W. T. R. Perera . 2016. “Prevalence of *Lactobacillus Bulgaricus* and *Streptococcus Thermophilus* Stability in Commercially Available Yoghurts in Sri Lanka.” Asian Journal of Medical Sciences 7, no. 5: 97–101.

[vms370233-bib-0048] Sah, B. N. P. , T. Vasiljevic , S. McKechnie , and O. N. Donkor . 2014. “Effect of Probiotics on Antioxidant and Antimutagenic Activities Of Crude Peptide Extract From yoghurt.” Food Chemistry 156: 264–270. 24629967 10.1016/j.foodchem.2014.01.105

[vms370233-bib-0049] Sah, B. N. P. , T. Vasiljevic , S. McKechnie , and O. N. Donkor . 2016. “Antibacterial and Antiproliferative Peptides in Synbiotic Yoghurt‐Release and Stability During Refrigerated Storage.” Journal of Dairy Science 99, no. 6: 4233–4242. 26995128 10.3168/jds.2015-10499

[vms370233-bib-0050] Settachaimongkon, S. , H. J. Van Valenberg , V. Winata , et al. 2015. “Effect of Sublethal Preculturing on the Survival of Probiotics and Metabolite Formation in Set‐Yoghurt.” Food Microbiology 49: 104–115. 25846920 10.1016/j.fm.2015.01.011

[vms370233-bib-0051] Shori, A. B. , G. S. Aljohani , A. J. Al‐zahrani , O. S. Al‐sulbi , and A. S. Baba . 2022. “Viability of Probiotics and Antioxidant Activity of Cashew Milk‐Based Yogurt Fermented With Selected Strains of Probiotic *Lactobacillus* spp.” LWT 153: 112482.

[vms370233-bib-0052] Song, J. , X. Shi , X. Li , and J. Zheng . 2022. “Choline diet Improves Serum Lipid Parameters and Alters Egg Composition in Breeder Ducks.” Veterinary Medicine and Science 8, no. 4: 1553–1562. 35384400 10.1002/vms3.798 PMC9297749

[vms370233-bib-0053] Şimşek, B. , and İ. Gün . 2021. “Some Physicochemical, Rheological and Sensory Properties of Flavored Ice Cream.” Niğde Ömer Halisdemir Üniversitesi Mühendislik Bilimleri Dergisi 10, no. 2: 598–605.

[vms370233-bib-0054] Taha, S. , M. El Abd , C. De Gobba , M. Abdel‐Hamid , E. Khalil , and D. Hassan . 2017. “Antioxidant and Antibacterial Activities of Bioactive Peptides in buffalo's Yoghurt Fermented With Different Starter Cultures.” Food Science and Biotechnology 26, no. 5: 1325–1332. 30263666 10.1007/s10068-017-0160-9 PMC6049774

[vms370233-bib-0055] Terpou, A. , A. Papadaki , I. K. Lappa , V. Kachrimanidou , L. A. Bosnea , and N. Kopsahelis . 2019. “Probiotics in Food Systems: Significance and Emerging Strategies Towards Improved Viability and Delivery of Enhanced Beneficial Value.” Nutrients 11: 1591. 31337060 10.3390/nu11071591 PMC6683253

[vms370233-bib-0056] Terzioğlu, M. E. , A. Arslaner , and İ. Bakırcı . 2023. “Çilekle Zenginleştirilmiş Manda Yoğurdunun Kalite Karakteristikleri İle Yağ Asidi Kompozisyonu, Ace İnhibitör Aktivite ve Hmf İçeriği Bakımından İncelenmesi.” Gıda 48, no. 2: 381–393.

[vms370233-bib-0057] T.S.1330 . 1999. “Yoğurt Standardı.” In Türk Standartları Enstitüsü.

[vms370233-bib-0058] Turgut, T. , and S. Cakmakci . 2018. “Probiotic Strawberry Yogurts: Microbiological, Chemical and Sensory Properties.” Probiotics Antimicrobial Proteins 10, no. 1: 64–70. 28417292 10.1007/s12602-017-9278-6

[vms370233-bib-0059] Uğur, E. 2023. “Peynirlerden İzole Edilen Peptitlerin Ace‐İnhibitör ve Antioksidan Aktivitesi Üzerine Kullanılan Enzimin Ve Süte Uygulanan İşlemlerin Etkisinin Araştırılması.” PhD diss., Süleyman Demirel Üniversitesi.

[vms370233-bib-0069] Uğur, E. , and Z. Öner . 2023. “Effects of Coagulants on Peptide Profiles and ACE‐Inhibitory Activity in Ultrafiltered White (Beyaz) Cheese.” Journal of Food Composition and Analysis 123: 105622.

[vms370233-bib-0060] Vargas‐Ramella, M. , M. Pateiro , A. Maggiolino , et al. 2021. “Buffalo Milk as a Source of Probiotic Functional Products.” Microorganisms 9, no. 11: 2303. 34835429 10.3390/microorganisms9112303 PMC8620832

[vms370233-bib-0061] Wang, J. , T. Wu , X. Fang , and Z. Yang . 2019. “Manufacture of Low‐Fat Cheddar cheese by Exopolysaccharide‐Producing *Lactobacillus Plantarum* JLK0142 and its Functional Properties.” Journal of Dairy Science 102, no. 5: 3825–3838. 30827553 10.3168/jds.2018-15154

[vms370233-bib-0062] Yalçın, H. , and Z. Polat . 2023. “Investigation of Functional Properties and Bacterial Viability of Probiotic Yoghurt Produced With *Lactobacillus acidophilus* LA‐5 in Long‐Term (12 Weeks) Storage.” Van Veterinary Journal 34, no. 1: 1–6.

[vms370233-bib-0063] Younas, S. , M. A. Murtaza , M. S. Manzoor , et al. 2024. “Effect of Probiotic Incorporation on Physicochemical Attributes of Yoghurt During Storage and Influence on Cholesterol Assimilation.” Journal of Food Science 89, no. 2: 1243–1251. 38174813 10.1111/1750-3841.16898

[vms370233-bib-0064] Zhang, S. S. , Z. S. Xu , L. H. Qin , and J. Kong . 2020. “Low‐Sugar Yogurt Making by the Co‐cultivation of *Lactobacillus Plantarum* WCFS1 With Yogurt Starter Cultures.” Journal of Dairy Science 103, no. 4: 3045–3054. 32059863 10.3168/jds.2019-17347

[vms370233-bib-0065] Zhou, T. , R. Huo , L. Y. Kwok , et al. 2019. “Effects of Applying *Lactobacillus Helveticus* H9 as Adjunct Starter Culture in Yoghurt Fermentation and storage.” Journal of Dairy Science 102, no. 1: 223–235. 30343912 10.3168/jds.2018-14602

[vms370233-bib-0066] Zicarelli, L. 2004. “Buffalo Milk: Its Properties, Dairy Yield and Mozzarella Production.” Veterinary Research Communications 28: 127. 10.1023/b:verc.0000045390.81982.4d 15372941

